# Problematic Facebook use behavior and locus of control in physiotherapy students

**DOI:** 10.1186/s43161-021-00031-1

**Published:** 2021-08-11

**Authors:** Yesim Salik Sengul, Turhan Kahraman, Buse Ozcan Kahraman

**Affiliations:** 1 School of Physical Therapy and Rehabilitation, Dokuz Eylül University, Izmir, Turkey; 2 Department of Physiotherapy and Rehabilitation, Faculty of Health Sciences, Izmir Katip Celebi University, Izmir, Turkey

**Keywords:** Facebook, Internet, Addiction, Student, Physiotherapy

## Abstract

**Background:**

Problematic Facebook use is a broader umbrella for term addictive-like symptoms, and scarce self-regulation related to Facebook use reflecting social and personal problems, and many studies have suggested that it is associated with many psychosocial problems. Locus of control (LOC) is described as a personality trait developed through social learning theory. Recently, LOC has got attention from both the patient’s and clinician’s perspectives. In addition, higher external LOC is associated with problematic Internet use. The aim was to investigate whether problematic Facebook use is associated with LOC. Four hundred twenty-one university students were enrolled in this cross-sectional study. Problematic Facebook use was determined by the Bergen Facebook Addiction Scale, and the participants were divided into two groups as neutral users and problematic users. Locus of control was assessed using the Locus of Control Scale, including subscales of personal control, belief in chance, the meaninglessness of the effortfulness, belief in fate, and belief in an unjust world.

**Results:**

In total, 333 students were eligible for the study. There were 66 students with neutral Facebook use (19.8%), and the remaining (n=267) had problematic Facebook use (80.2%). No significant difference was observed in the demographic characteristic of neutral and problematic Facebook users (p>0.05). Problematic Facebook users had significantly higher scores on the meaninglessness of the effortfulness (p<0.001), belief in fate (p=0.019), and belief in an unjust world (p=0.004) compared to the neutral Facebook users.

**Conclusions:**

The results showed that the physiotherapy students having a problematic Facebook had significantly higher scores on the meaninglessness of the effortfulness, belief in fate, and belief in an unjust world compared to neutral Facebook users. All these negative thoughts might be a problem both for students themselves and their future patients.

## Background

Problematic Facebook use is a broader umbrella term for addictive-like symptoms, and scarce self-regulation related to Facebook use reflecting in social and personal problems, and many studies have suggested that it is associated with many psychosocial problems [1]. The outbreak of COVID-19 has significantly restricted people’s everyday life and contributed to enhanced social media use [2], and people, especially students, have started to use social media since they are at home and hostels and have more free time more than before. During the COVID-19, our personal and professional lives interfered (mixed) through Facebook, Twitter, and Instagram [3]. Also, a study showed that 50.8% of the university students reported usually obtaining their information about COVID-19 from social media such as Facebook, Twitter, and Instagram [4]. It was shown that 47.8% of students reported they were watching new stories about COVID-19 for 1–3 h a day and 4.8% for more than 6 h [4]. In addition, education in universities in Turkey and many countries have currently begun digital instruction and distance learning [5]. In addition to get information about the latest news and leisure time engagements, many university students use social media networks as an instrument to disseminate information and to communicate in their academic and research activities [6]. Overall, many external factors of life can influence Facebook and social media use.

Locus of control (LOC), connected to individuals’ characteristics, is described as a personality trait developed through social learning, and it has two main categories as internal and external LOC [7]. Internal LOC is an indicator of individual actions and initiatives, while luck, fate, and other persons affect external LOC. Some studies have indicated that individuals who have external LOC spend more time on academic activities for the same job than individuals with internal LOC, and also, these persons have better results at school and competitions [8, 9]. This situation is associated with personality traits as low self-esteem, depressive mood, feeling of loneliness, and passive personality structure [8].

A study conducted on 1245 college students in China showed that excessive use of a mobile phone-based messaging and social networking application is associated with external LOC [10]. Thus, people who believe that external factors cause events in their lives tend to overuse this application. Externals perceive less control of their environment and hence fail to regulate their social networking application and behavior [10]. Another Chinese study also revealed that more lonely, unhappy, and externally controlled students were more likely to be engaged in online social interaction [11].

A person’s control beliefs over many fields of life are an important determinant. Health LOC, another version of LOC, was developed to evaluate the patient’s belief in the health and physiotherapy field [12–15]. Turkish patients had a high chance and external beliefs, and these factors were related to increased disability levels, decreased quality of life, and increased pain severity [14, 15]. Most physiotherapy professionals deal with patients with chronic pain for many treatment sessions. Chronic pain has a complex mechanism, leading to impairments in patients’ lives as a biopsychosocial being. In the literature, chronic pain has been reported to cause chronic pain behavior, depression, fear of activity, and anxiety [16]. Patients with chronic pain need more external support, especially from health practitioners like life coaches [13]. The biopsychosocial model should be adapted to clinical practice since recent evidence suggests that psychosocial risk factors lead to prolonged disability and increased pain levels [17]. Patients with a developing long-term disability risk and higher levels of catastrophizing or depression might have shown more significant reductions in disability if physiotherapists’ attitudes and beliefs changed [18]. The importance of behavior and positive belief of physiotherapists in managing patients with chronic pain is well known [19]. Therefore, investigating the association between problematic Facebook use and a person’s control beliefs in physiotherapist candidates emerges as an important topic. The present study aimed to investigate the association between problematic Facebook use and LOC.

## Methods

### Participants

This cross-sectional study sample consisted of 421 students from School of Physical Therapy and Rehabilitation, Dokuz Eylül University, Izmir, Turkey. All the volunteer students from all the classes were included in the study. Non-Facebook users and students with missing data in their questionnaires were excluded. The researchers explained the study to the students in a course, and the questionnaires were distributed. The students were asked not to exchange ideas with each other while filling out the questionnaires.

The study protocol was approved by the Noninvasive Research Ethics Board of Dokuz Eylül University (registration number: 2757-GOA), and all participants gave informed consent for the use of their data in the study. The required sample size was calculated as 194 with type I error rate = 0.05, type II error rate = 0.20, and expected correlation coefficient = 0.20 as a study reported that the correlation coefficients between LOC and internet addiction were ranged from − 0.13 to 0.27 among students [20].

### Study outcome measures

A simple data collection form was prepared to gather demographic characteristics and Facebook use time. This form had questions asking participant’s gender, age, year of study, and Facebook use time (hours/day). After filling out this form, the participants filled out the Locus of Control Scale and Bergen Facebook Addiction Scale.

### Locus of Control Scale

The Locus of Control Scale is a valid and reliable LOC measure with a more comprehensive and simple factor structure than the previous Turkish version of Rotter’s Internal-External Scale for the Turkish population [21]. The scale consists of 47 items answered in a 5-point Likert-type scale with that increasing scores indicate external control [21]. The LOC scale is divided into five subscales: personal control, belief in chance, the meaninglessness of the effortfulness, belief in fate, and belief in an unjust world. The LOC scale had a high internal consistency (ICC=0.92), and five domains explained 50.63% of the variance [21].

### Bergen Facebook Addiction Scale

The Bergen Facebook Addiction Scale (BFAS) is a valid and reliable instrument for measuring Facebook addiction [22]. It comprised 18 items, three for each of the six core features of addiction: salience, mood modification, tolerance, withdrawal, conflict, and relapse. Each item was scored on a 5-point Likert-type scale where higher scores indicating greater Facebook addiction [22]. The internal consistency of the scale is 0.83, and a 3-week test-retest reliability is 0.82 [22]. This scale was translated into Turkish, and this adapted version was shown to be valid and reliable [23]. The Turkish version of BFAS had high internal consistency (ICC=0.93), and the model fit indices of the confirmatory factor analysis applied to determine the construct validity of the scale were adequate [23]. The cutoff points of BFAS were accepted as 20, and the participants were divided into two groups as neutral Facebook use and problematic Facebook use. Further, the problematic Facebook use was categorized as mild (21–40), moderate (41–69), and severe Facebook addiction (70–90).

### Statistical analysis

Data were analyzed using the IBM SPSS for Windows software (Version 25.0. Armonk, NY: IBM Corp.) The normal distribution of data was investigated using visual and analytical methods (Kolmogorov-Smirnov test) to determine. Due to the non-normal distribution of age, LOC subscales, and BFAS, non-parametric statistics were conducted. Descriptive analyses were presented as median values and interquartile range (IQR) for continuous variables and number and percent for nominal variables. Comparison between the students having neutral and problematic Facebook use was conducted using the Mann-Whitney U test. In addition, a logistic regression analysis was used to analyze the predictors of problematic Facebook use. The level of statistical significance was set at *p*<0.05.

## Results

Ninety-one students were excluded from the study because of no Facebook use (n=43) and incomplete data (n=48). Therefore, 333 students’ data were analyzed. The sample consisted of 164 males (49.2%) and 169 females (50.8%); median age = 22 (IQR = 21–23) years. There were 66 students with neutral Facebook use (19.8%), and the remaining (n = 267) had problematic Facebook use (80.2%). There were 161 (48.3%) students with mild Facebook addiction, 99 (29.7%) with moderate, and 7 (2.1%) with severe Facebook addiction. No significant difference was observed in the basic sociodemographic characteristic of neutral and problematic Facebook users (p>0.05). Median Facebook time was significantly higher in the problematic Facebook users (p<0.001) (Table 1).

**Table 1:** Characteristics of the participants

	Total sample (n=333)	Neutral Facebook users (n=66)	Problematic Facebook users (n=267)	p
**Gender**				
**Female**	169 (50.8)	36 (54.5)	133 (49.8)	0.496
**Male**	164 (49.2)	30 (45.5)	134 (50.2)	
**Age (years), median (IQR)**	22 (21–23)	22 (20–23)	22 (21–23)	0.093
**Year of study**				
**1st (Freshmen)**	47 (14.1)	13 (19.7)	34 (12.7)	0.490
**2nd (Sophomores)**	71 (21.3)	12 (18.2)	59 (22.1)	
**3rd (Juniors)**	120 (36.0)	24 (36.4)	96 (36.0)	
**4th (Seniors)**	95 (28.5)	17 (25.8)	78 (29.2)	
**Facebook use time (hours/day)**	1 (0.5–1)	0.5 (0.25–1)	1 (0.5–2)	<0.001*
**BFAS**	33 (22–45)	18 (18–19)	37 (28–49)	<0.001*

*p<0.05

Data values are number (percent) unless otherwise stated

*BFAS*, Bergen Facebook Addiction Scale; *IQR,* interquartile range

**Fig. 1:**
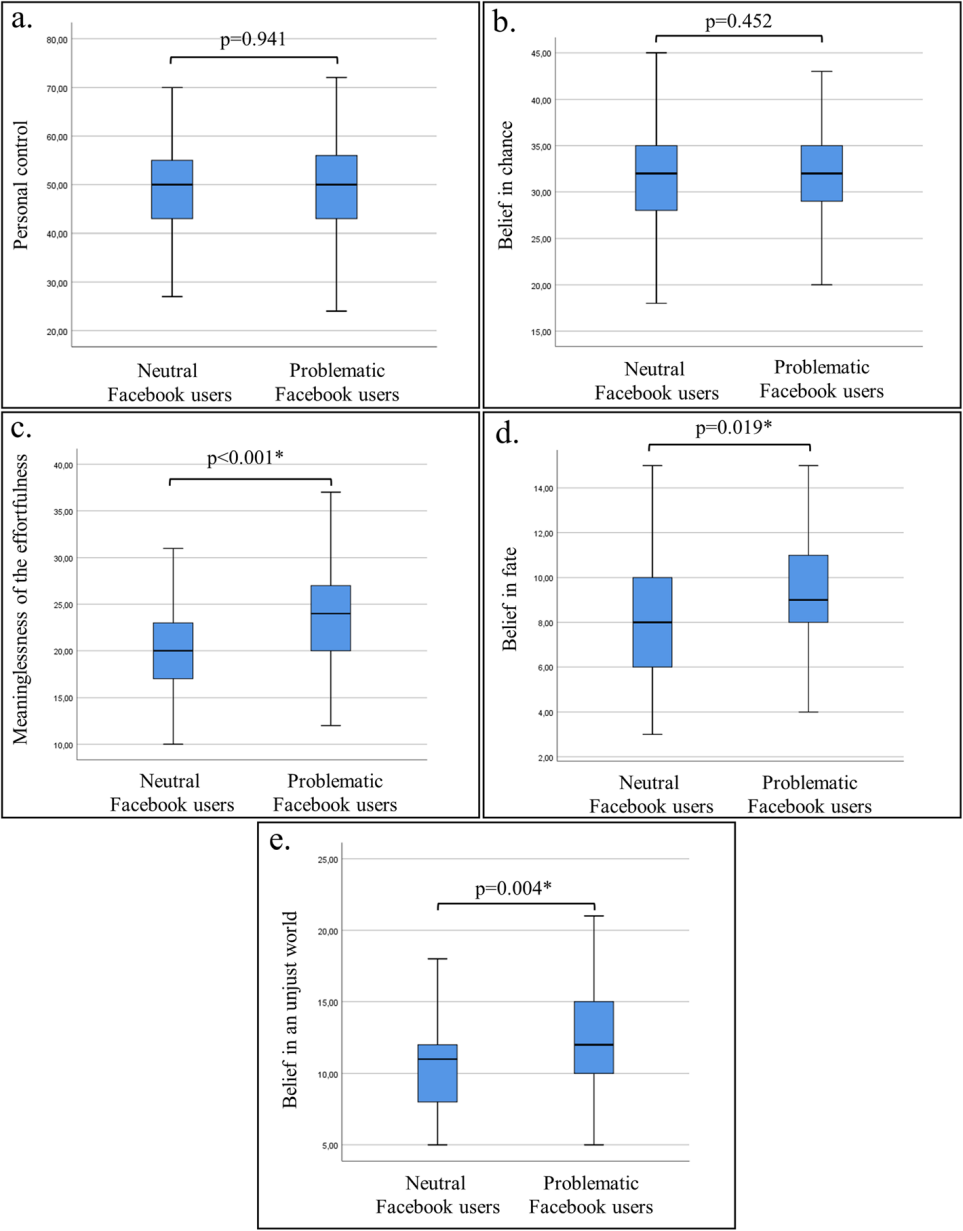
Comparison of the locus of control domains (**a** personal control, **b** belief in chance, **c** meaninglessness of the effortfulness, **d** belief in fate, **e** belief in an unjust world) between neutral vs. problematic Facebook users. Medians are shown by lines in the center of the box-plots; the 25th–75th percentiles are indicated by the boxes and the range by the whiskers

Problematic Facebook users had significantly higher scores on the meaninglessness of the effortfulness (p<0.001), belief in fate (p=0.019), and belief in an unjust world (p=0.004) compared to the neutral Facebook users (Table 2). Logistic regression analysis revealed that meaninglessness of the effortfulness was the only significant predictor of the problematic Facebook use with an odds ratio of 1.12. Figure 1 presents comparisons of the locus of control domains between neutral vs. problematic Facebook users.

**Table 2:** Comparison of the locus of control domains between neutral vs. problematic Facebook users

Locus of control domains	Total sample (n=333)	Neutral Facebook users (n=66)	Problematic Facebook users (n=267)	p	OR	95% CI
**Personal control**	50 (43–56)	50 (43–55)	50 (43–56)	0.941	1.0	0.97 to 1.03
**Belief in chance**	32 (28–35)	32 (27.5–35.5)	32 (29–35)	0.452	0.97	0.91 to 1.04
**Meaninglessness of the effortfulness**	23 (19–26)	20 (17–23.25)	24 (20–27)	<0.001*	1.12*	1.03 to 1.20
**Belief in fate**	9 (8–11)	8 (6–10.5)	9 (9–11)	0.019*	1.08	0.97 to 1.21
**Belief in an unjust world**	12 (9–14)	11 (8–12.25)	12 (10–15)	0.004*	1.05	0.95 to 1.17

*p<0.05

Data values are median (interquartile range)

*OR*, odds ratio; *CI*, confidence interval

The BFAS scores significantly correlated with personal control (rho=0.16, p= 0.008), meaninglessness of the effortfulness (rho=0.31, p<0.001), and belief in an unjust world (rho=0.20, p=0.001) (Table 3). However, no significant correlation was observed between the BFAS and belief in chance and belief in fate (p>0.05) (Table 3).

**Table 3:** Correlations between the BFAS and LOC subscale scores in the students with problematic Facebook (n=267)

	BFAS	LOC-personal control	LOC-belief in chance	LOC-meaninglessness of the effortfulness	LOC-belief in fate
**LOC-personal control**	0.16 (0.008*)	-			
**LOC-belief in chance**	− 0.02 (0.811)	0.12 (0.047*)	-		
**LOC-meaninglessness of the effortfulness**	0.31 (<0.001*)	0.03 (0.577)	0.38 (<0.001*)	-	
**LOC-belief in fate**	0.05 (0.377)	− 0.14 (0.02*)	0.16 (0.009*)	0.22 (<0.001*)	-
**LOC-belief in an unjust world**	0.20 (0.001*)	0.03 (0.589)	0.30 (<0.001*)	0.57 (<0.001*)	− 0.01 (0.951)

*p<0.05

Values are presented as Spearman’s rho (p)

*BFAS*, Bergen Facebook Addiction Scale; *LOC*, locus of control

## Discussion

In the present study, the association between problematic Facebook use and LOC has been investigated. The results showed that the problematic Facebook users had significantly higher scores on the meaninglessness of the effortfulness, belief in fate, and belief in an unjust world compared to the neutral Facebook users. Besides, increased Facebook addictive behavior was significantly associated with increased external control in personal control, meaninglessness of the effortfulness, and belief in an unjust world. Among the investigated variables, the meaninglessness of the effortfulness was the only significant predictor of problematic Facebook use.

Facebook use is an important indicator and a specific form of Internet addiction. The Internet and Facebook addiction level have been high, especially among students in health-related studies [24–26]. Studies on various undergraduate health students showed that the physiotherapy group had higher Internet addiction [26, 27]. Among the present study participants, most of the students had a mild and moderate level of Facebook addictive behavior determined by the BFAS.

The findings of the current study indicated that the problematic Facebook users believe that if something happens, it will happen, in other words, meaninglessness to try. In addition, they believe fate has a significant role in human life, and powerful people determine the direction of human life. All these negative thoughts might be a problem both for the students themselves and their future patients.

Self-control is crucial for addictive behavior and connected with self-esteem and managing stress. Firat [28] found a negative relationship between Facebook addiction and self-control. Consequently, as indicated in most of the studies above, Internet addiction, including Facebook, leads to impaired inhibitory control processing, decreased social interactions, and depression [29]. Researchers have indicated the importance of perceived control in managing stress and academic achievement [9, 30]. LOC was defined as individuals’ attributions for the causes of outcomes [7]. If the behavior is viewed as determined by one’s influence, the LOC is labeled internal. Individuals with a high internal LOC are better psychologically adjusted than those having higher external LOC [10]. On the contrary, if the expectancy for the behavior is perceived as being outside one’s control, it is considered external LOC, highlighting individuals’ point of view for external factors such as fate, luck, or powerful others to be responsible for an outcome [10]. Internal LOC was related to academic success, self-esteem, and stress management [31, 32]. Internet addiction was negatively associated with internal LOC [20].

Similarly, Iskender et al. [33] found that Internet addiction was associated with less social self-efficacy and internal academic LOS and high external academic LOS. The present study results are consistent with the finding that fate beliefs like meaninglessness of the effortless, belief in fate, and belief in an unjust world in the LOC scale were associated with Facebook problematic use. Another study also reported that more lonely and unhappy students with high external LOC were more likely to be engaged in online social interaction [11]. These findings have supported that problematic Facebook use is associated with external factors such as chance and fate beliefs.

Patients’ benefits from the therapy might be related to the physiotherapists’ attitudes and beliefs [18]. Also, the importance of behavior and positive belief of physiotherapists in managing patients with chronic pain is well known [19]. The current study is important by providing information about the association between problematic Facebook use and a person’s control beliefs in physiotherapist candidates. Since our study has a cross-sectional design, we cannot show a cause-effect relationship between the problematic Facebook use and LOC. However, reducing problematic Facebook use may help improving students’ LOC; thus, they can provide much effective services to their future patients. However, this hypothesis should be proved with the longitudinal studies. In addition, since technology and its innovation are super fast in connecting the life of new generations, but its results in different cultures should be investigated in future studies.

There were several limitations to the present study. Firstly, it had a cross-sectional design; therefore, its results cannot imply causation between problematic Facebook use and LOC. Additionally, Facebook use characteristics could have changed over the years, and the cross-sectional findings cannot be enough to reflect this possibility. Therefore, longitudinal studies are highly warranted. Second, we only investigated Facebook use; however, it is evident that other social media platforms, especially WhatsApp and Instagram, are so frequent. Future studies should investigate their effects on LOC. Lastly, since the study sample was recruited from a single state university, the study’s generalizability of findings was limited. Further multicenter studies including students from other private and state universities and different departments would be more informative and representative of the general population.

## Conclusion

Problematic Facebook use was associated with higher external LOC in physiotherapy students. The students having a problematic Facebook use behavior believe meaninglessness of the effortfulness, fate, and an unjust world more than those having a neutral use. The meaninglessness of the effortfulness was the only significant predictor of problematic Facebook use. These negative thoughts might be a problem both for the students themselves and their future patients. The current study’s findings indicate an increasing trend for social network use of physiotherapy students who will treat increased psychosocial distress or chronic disability of patients with chronic pain.

## Data Availability

Data will be shared on specific request to the author depending upon the nature and purpose of the requirement.

## Abbreviations

LOC: Locus of control
BFAS: Bergen Facebook Addiction Scale
IQR: Interquartile range
